# The feminist appropriation of pregnancy testing in 1970s Britain

**DOI:** 10.1080/09612025.2017.1346869

**Published:** 2017-07-11

**Authors:** Jesse Olszynko-Gryn

**Affiliations:** Department of History and Philosophy of Science, University of Cambridge, Cambridge, UK

## Abstract

This article restores pregnancy testing to its significant position in the history of the women’s liberation movement in 1970s Britain. It shows how feminists appropriated the pregnancy test kit, a medical technology which then resembled a small chemistry set, and used it as a political tool for demystifying medicine, empowering women and providing a more accessible, less judgmental alternative to the N.H.S. While the majority of testees were young women hoping for a negative result, many others were older, menopausal women as well as those anxious to conceive. By following the practice of pregnancy testing, I show that, at the grassroots level, local women’s centres were in the vanguard of not only access to contraception and abortion rights, but also awareness about infertility and menopause.

Sue Jones was on her way to be interviewed for the Bolton Women’s Liberation Group Oral History Project when, passing the back of the town’s historic Market Hall, she thought to herself, ‘“I must tell them about the pregnancy testing” because we started off,’ she later explained at the interview:

opposite there, and I think it’s a nightclub now, or part of a nightclub. A Holiday Inn! That’s what it is. It used to be the old Co-op funeral parlour, and that’s where we used to do the testing. We were given the use of the premises free I think, and it was a *dump*, but that’s where we did it—it was very central, it was very good and we did it every Saturday morning, we did it for quite a long time.

By the time of the interview, some thirty-seven years after she had joined the group as a young mother in 1972, Jones did not remember where they ‘got the kit from,’ but she did remember that ‘you had to do one or two things with the kit; it wasn’t like the ones you get from Boots now where you just wee on a stick!’ ‘We did pregnancy testing,’ she told the interviewer, ‘and it was very important to us in those days.’^1^

Histories of the British women’s liberation movement occasionally mention free pregnancy testing and ‘post-test counselling’ in passing, typically in connection to women’s health centres, but little is known about the practices or politics of this ‘vital service’.^2^ Directly inspired by the American women’s health movement and its self-help manual *Our Bodies, Ourselves* (1971), British feminists launched their own critique of medical sexism.^3^ From the First National Women and Health Conference in Sheffield in 1974 to Sue O’Sullivan’s regular health column in the magazine *Spare Rib* (1972–1993), a newly frank emphasis on the female body and women’s health ran through the British movement.^4^ Free access to health care and the ‘iconic status’ of the N.H.S., however, meant that (socialist) feminists generally aimed at reforming the ‘much-valued’ system from within.^5^ Whereas most attempts to set up feminist alternatives to the N.H.S. failed, more limited local services such as pregnancy testing flourished.^6^ Alongside the more canonical activities of cervical self-examination and menstrual extraction, pregnancy testing was, on both sides of the Atlantic, part of a broader ‘strategy of empowering women with information and access to technology.’^7^

In Britain, the grassroots pregnancy testing offered by women’s groups in the 1970s built on a longer tradition of feminist activism and the involvement of women’s voluntary organisations in always-controversial services around fertility control. These went back at least to 1921, when Marie Stopes and her husband opened the first Mothers’ Clinic in working-class Holloway, North London.^8^ Following Stopes, the National Birth Control Council, later the Family Planning Association (F.P.A.), established an extensive network of clinics that was absorbed into the N.H.S. in 1974, when contraception became freely available to unmarried women.^9^ From the late 1960s, Brook Advisory Centres provided contraception to minors, and the London-based Pregnancy Advisory Service (P.A.S.) as well as the Birmingham Pregnancy Advisory Service (B.P.A.S.) managed a network of non-profit abortion clinics.^10^ Dependent on alliances between laywomen and sympathetic doctors, these organisations filled gaps in N.H.S. provision and existed in a relationship of both tension and collaboration with the medical establishment and welfare state.

Projects such as *Sisterhood and After* and the digitisation of *Spare Rib* have recently provided fresh resources with which to enrich the history of the women’s liberation movement.^11^ In this article I marshal a range of previously unused materials to show how British feminists managed to appropriate the pregnancy test, which then resembled a small chemistry set. As with the plastic speculum and ‘Del-em’ menstrual extraction device, they used the test kit as a political tool for demystifying medicine and empowering women, bringing the medical technology into the domestic sphere and realigning it with the feminist politics of the day.^12^ As in America, where pregnancy testing was similarly widespread in 1970s, women’s groups in cities around Britain established local services as a means of facilitating access to abortion, contraception and information.^13^ They expected to serve mainly young women dreading pregnancy. But as the testers soon found out, while a negative result came as a relief to most testees, including not only girls and young women, but also older, menopausal women, it disappointed numerous others who were trying to conceive. The ‘many faces’ of pregnancy testing surprised some activists, who later saw themselves as having been in the vanguard of not only access to contraception and abortion rights, but also awareness about infertility and menopause. I argue that, while infertility and menopause never achieved the status of abortion or contraception at the level of national campaigning, at the grassroots level, feminists engaged with these other aspects of women’s reproductive lives more than histories of either the liberation movement or infertility generally acknowledge.

## Pregnancy ‘diagnosis’, abortion, and the N.H.S.

Women have long relied on bodily signs such as a missed menstrual period or morning sickness to self-diagnose pregnancy.^14^ By the early twentieth century, some working-class women continued to offer their urine for visual inspection to the ‘water doctor’, but the medical encounter was increasingly mediated by the laboratory, including for pregnancy testing.^15^ Between the late 1920s and the mid 1960s, laboratory workers injected women’s urine into living animals—first mice and rabbits, then frogs and toads—to ‘diagnose’ pregnancy. If present in sufficiently high concentration in the patient’s urine sample, the ‘pregnancy hormone’ today known as hCG (human chorionic gonadotrophin, the same molecule later detected by home tests) triggered physiological changes in the animals, which reliably constituted a ‘positive’ result. Crucially, pregnancy testing was, in this period, a diagnostic service for medical professionals only; the only way a woman could obtain the result of a laboratory test was from her doctor. A few specialised centres and most hospitals, but not doctors’ surgeries, were equipped for pregnancy testing. G.P.s would post a patient’s urine sample to a lab and it could take a week or more for the result to come back.^16^

From the late 1940s, pregnancy testing was made freely available on the N.H.S., but only for medical reasons; doctors rejected demand from so-called curiosity cases: healthy married women likely to have an uneventful pregnancy.^17^ Doctors’ requests for all kinds of laboratory investigations, including pregnancy tests, doubled from around 22 million in 1961 to 45 million in 1971, straining a health system that was facing a major financial crisis by the mid 1970s.^18^ The widely publicised birth defects caused by thalidomide and rubella in the early 1960s as well as the somewhat later campaigns around smoking in pregnancy and fetal alcohol syndrome incentivised women, including those hoping to conceive, to get tested earlier and in greater numbers than any previous generation.^19^

The meticulously kept records of a rural G.P. interviewed by feminist sociologist Ann Oakley in the early 1980s show that he ordered pregnancy tests for only 1.3% of his female patients in the late 1940s and 38.8% in the late 1970s, a thirty-fold increase in three decades.^20^ Many G.P.s, however, disapproved of ‘social’ pregnancy testing as an abuse of the already overstretched service. From the mid 1960s, by which time mass-produced immunological test kits had supplanted living animals, commercial labs served women directly, not as ‘patients’, but as ‘clients’. So too did pharmacists as well as branches of the F.P.A., Brook, B.P.A.S. and P.A.S. The thriving non-medical market for pregnancy testing paved the way for Britain’s first do-it-yourself test kit, Predictor, in 1971.^21^ By then, some two-thirds of all women had heard of the once taboo subject of pregnancy testing.^22^

Predictor was available through the 1970s from most pharmacies except Boots, Britain’s largest and historically conservative chain, but self-testing was not universally embraced overnight.^23^ In 1974, the consumers’ watchdog *Which?* advised going to one’s G.P. in the first instance and warned that ‘clumsy’ users could end up wasting ‘almost £2’ on the ‘do-it-yourself’ kit. It also reviewed free testing from B.P.A.S. branches alongside commercial labs and family planning clinics, which charged between £1.50 and £3 (perhaps ten times as much in today’s money).^24^ Today this might not seem like much, but for a young student in Leeds in the late 1960s, a commercial pregnancy test cost as much as a week’s rent.^25^ Nevertheless, in the early 1970s, pregnancy testing was available from a variety of sources in a variety of forms.^26^ To this mix was added free or at-cost pregnancy testing by women’s liberation groups around the country.

Feminist pregnancy testing was closely connected to grassroots activism and national campaigns for access to contraception and abortion. Before the landmark conference at Ruskin College in Oxford launched the women’s liberation movement on 27 February 1970, two pieces of legislation from 1967 had already liberalised access to contraception and abortion: the National Health Services (Family Planning) Act and the Abortion Act, the latter coming into force in England, Wales and Scotland on 27 April 1968. From the formation of the Society for the Protection of the Unborn Child (S.P.U.C.) in 1967 and splinter group LIFE in 1970, the anti-abortion movement redirected feminist campaigns towards reproductive rights under the N.H.S.^27^ ‘Free contraception and abortion on demand’ was one of the four demands adopted in 1970 in preparation for the Women’s Day march on 8 March 1971—alongside ‘equal pay now’, ‘equal education and job opportunities’, and ‘free 24-hour childcare’. Abortion became ‘almost the definitive issue’ of the British women’s movement in the 1970s, with massive demonstrations against the anti-abortion bills of James White (1975) and John Corrie (1979), among others.^28^

Meanwhile, the relationship between pregnancy testing and abortion came under scrutiny when Sir Keith Joseph, the Conservative Secretary of State known for his contentiously Malthusian views, established a committee of enquiry into the working of the Act in 1971.^29^ Chaired by Justice Elizabeth Lane, England’s first female high court judge, the committee’s three-volume report, published in April 1974, stated that ‘the safest time for termination is before the twelfth week of pregnancy’ and stressed the importance of ‘expeditious diagnosis of pregnancy […] both from the woman’s point of view and that of her medical care.’^30^ The report, which strongly upheld the Act, further found that:

the most satisfactory method of obtaining a test is through a general practitioner, although it is a disadvantage that, if he [sic] sends the sample to a NHS laboratory for testing, it may be several days before the result can be communicated to the patient.^31^

The report recommended ‘that NHS laboratories should make arrangements, where they do not already exist, for pregnancy testing on all referrals from doctors and for prompt communication of the results’, and ‘that the Health Departments should consider the feasibility of providing pregnancy testing facilities in addition to those services already provided at NHS hospitals.’^32^

In 1972, the Women’s National Commission, an umbrella body for women’s organisations set up by the government in 1969, made headlines when its report to the Lane Committee urged a ban on ‘unreliable’ self-testing kits, a suggestion echoed by B.P.A.S. in 1974, and recommended ‘that private pregnancy testing services should be licensed’.^33^ The Women’s Abortion and Contraceptive Campaign (W.A.C.C.), formed in 1971 by feminists to oppose efforts to restrict the Act as well as to shift decision-making power from doctors to women,^34^ also weighed in on pregnancy testing, but from a different standpoint. As part of evidence presented to the Lane Committee—including women’s abortion narratives and a manifesto demanding free contraception, no forced sterilisation, and abortion on demand—W.A.C.C., later subsumed under the National Abortion Campaign (N.A.C.),^35^ pledged to ‘provide community pregnancy testing and pregnancy advisory centres’ to enable women to ‘find out quickly and easily when they are pregnant’ and ‘talk to others face to face or over the telephone about pregnancy, abortion and contraception and the many other related areas.’ Grassroots pregnancy testing thus emerged as the campaign’s first step towards demonstrating the sort of N.H.S. services that women ‘really need rather than having to accept the services the medical profession and government thinks we need.’^36^

**Figure 1.**
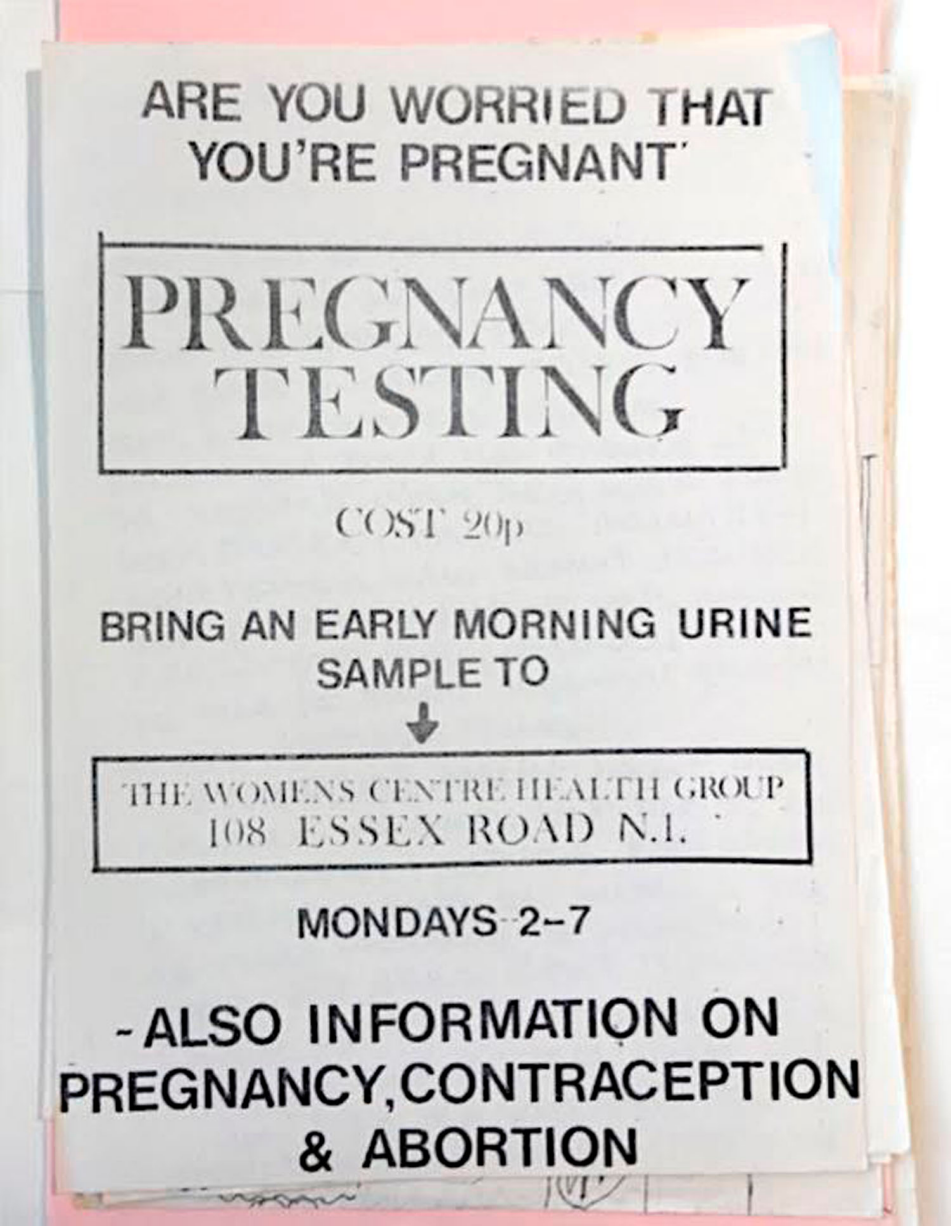
Leaflet produced by the Essex Road Women’s Centre, *c*.1973. Records of Essex Road Women’s Centre, Women’s Library @ LSE, London (5ERC/5/1).

Such services rapidly proliferated in the early 1970s. Ealing feminists, as Ann Oakley later recalled, ‘acquired the easy skill of pregnancy-testing,’ which they ‘offered gratuitously for a short while to the women of Acton’. In her account, pregnancy testing was one of several activities alongside cervical self-examination, organising jumble sales, writing ‘“where we are at” papers’ and producing copies of *Shrew*, ‘the aptly named women’s liberation paper’.^37^ Local groups in Bolton, Bradford and Merseyside were soon offering free pregnancy tests.^38^ The health group set up by the Essex Road Women’s Centre in Islington ‘did pregnancy testing’, provided ‘a woman doctor for advice sessions’, kept tabs on local doctors’ ‘treatment of women’ and provided ‘information on abortion facilities’ (Figure 1).^39^ By the time the Lane Committee’s report was published in April 1974, a disapproving ‘feminist view’ in *Spare Rib* could dispute its proposal to consider pregnancy testing services for licencing on the grounds that women’s groups in ‘many cities’ were already:

doing pregnancy testing the way we want it done: with easy access, immediate results, and either free or for the cost of the test alone. The tests are done by women who understand and are sympathetic to the feelings and needs of the women using the services, and WACC activists are cynical about the ability of the NHS, as it is presently structured, to provide a similar service.^40^

## Getting started

Biographical evidence of women’s centres is ‘fragmentary’ and their history ‘underdeveloped’,^41^ but archival records are plentiful and newsletters, mission statements and oral-history interviews make it possible to follow the practice of activist pregnancy testing in the early 1970s. The Cambridge Pregnancy Advisory Group (C.P.A.G.), for which particularly informative documentation exists, was started by women returning from the first national W.A.C.C. conference in Liverpool in January 1973 as well as women and men who were working on a survey of contraceptive use by Cambridge students. Membership fluctuated between eight and sixteen people, mostly women, and turnover was high with testers regularly leaving Cambridge or moving their energies elsewhere in the movement. Clients, including students, came from all around the city and surrounding villages, sometimes accompanied by a mother or partner. New recruits, including grateful testees such as Lucy King, joined on a regular basis. Mary Bernard, a native of Montreal who came to England in 1964, decided to get involved because of her ‘gruesome’ experience of illegal abortion in the Canadian maritime province of New Brunswick.^42^

C.P.A.G. started off with a loan of £25 from the local Women’s Liberation Group, which was still outstanding two years later. They held sessions twice a week, on Wednesday evenings and Saturday mornings, to accommodate increasingly typical patterns of women’s work, study and childcare, in the corner of a shabby room in a centrally located residential house rented by the women’s group, at 48 Eden Street. Crucially, they also had access to ‘running water, a toilet and a fridge to store the chemicals in.’ Women did not ‘seem to mind being tested by men,’ and C.P.A.G. encouraged the presence of (supportive) male partners ‘because contraception involves them as well as women.’^43^ This policy eventually became ‘out of synch with the rest of the women’s movement’ and led to a stand-off with separatist feminists who blockaded the women’s centre, which by then had moved to Adam and Eve Street. C.P.A.G. promptly relocated again, this time for good, to Centre 33, a nearby Brook clinic.^44^

**Figure 2.**
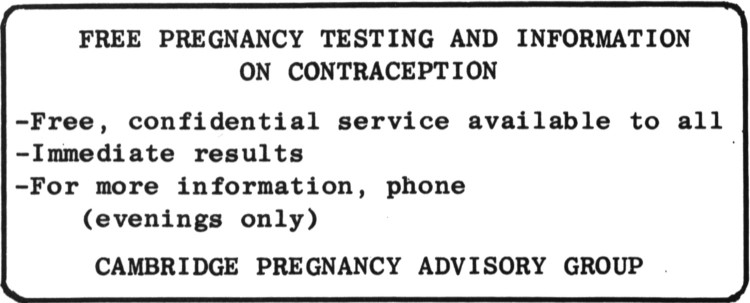
C.P.A.G. publicity card courtesy of Mary Bernard.

Compared with other activities, pregnancy testing did not cost much, but it still needed money to buy test kits, pay the rent and print leaflets for advertising. Notices in the testing room publicised the 20p cost of each test and invited donations. Many testees overpaid and C.P.A.G. generated extra funds by selling feminist literature and also received donations from student unions. By May 1975 they had negotiated promises of financial help from the Regional Health Authority on the grounds that they were ‘doing their work for them (since pregnancy tests are supposed to be available under the NHS)’.^45^ C.P.A.G. advertised weekly in the personal column of the local newspaper and displayed stickers, cards and posters in town (Figure 2). The stickers did ‘very well on toilet doors’, sometimes staying up for years at a time. The local Citizens Advice bureau, Samaritans and Local Authority Family Planning Office promoted the service and, before long, word of mouth also played a significant role. By 1975 the group was doing around 400 tests every year.

C.P.A.G. held a meeting every Wednesday after testing to go over the previous week’s tests, ‘pool information and expertise’, and ‘discuss any problems’ that came up. They kept ‘simple records’ of every test, which consisted of:

First name (if willing); date of LMP [‘last menstrual period’, used by doctors to estimate the gestational age of the fetus], average cycle length, no. days late; early morning sample or not; results; drugs taken; contraceptive if used; whether wants to be pregnant or not; confirmation of result; how they heard of us; names of testers.^46^

Members kept up to date by reading the *British Medical Journal* and *Lancet* as well as the medical correspondence in daily newspapers. They also maintained a card index on ‘attitudes of local G.P.s to abortion, contraception and women in general, whether married, unmarried, or very young’, which they then used to help a woman decide between going to a family planning clinic or her own G.P. for contraceptive or abortion advice. Most local G.P.s agreed to accept the group’s results without a retest and C.P.A.G. supplied each testee with a result card which she could ‘show […] her doctor if necessary.’ They also tried to use the two minutes afforded by the test to ‘find out whether the woman wants to have a child,’ in order to help decide whether it was ‘relevant to raise the subject of abortion.’ It was ‘very important,’ they asserted, ‘not to make any assumptions about women’s attitudes and certainly not to pressure them towards abortion but simply to help them see what is involved in the decision either way.’ Finally, they also tried to explain their belief ‘that women must have control over their bodies so that they can control their lives’ as well as how the test worked in order to ‘dispel some of the medical “mystique” surrounding pregnancy testing.’^47^

In Bristol, where comparably detailed records exist, a pregnancy testing service was likewise set up by women affiliated to W.A.C.C. They operated out of the local women’s centre, which opened in March 1973 in the one-room basement kitchen of Ellen Malos’s house in suburban Redland. The centre advertised its address and telephone number through the local press and radio as a ‘woman’s information service’ and similarly offered pregnancy testing twice a week, on Thursday evenings and Saturday mornings. The rota was ‘wommaned’ by trained volunteers, two or three at a time, who also offered advice on contraception and abortion, collected information about ‘conventional women’s health services in the Bristol area,’ and liaised with the local Brook clinic.^48^ Despite the availability of pregnancy testing through local authority clinics, the Bristol group hoped to ‘attract women who might be put off by the “official” institutions.’^49^ In this they seem to have succeeded in meeting a significant demand. By January 1974, they were performing three to five tests every week and, by April, as many as a dozen.^50^ By 1976, the group was doing around 500 tests every year, on a par with the local hospital’s 800 tests on samples sent by family planning clinics.^51^

The Bristol group tried to provide a ‘friendly informal atmosphere’ for women of ‘all ages and social backgrounds’, some of whom travelled ‘great distances, and certainly from all over Bristol and the surrounding rural areas.’ In a ‘Dear Sister’ open letter, from November 1974, they explained that their service filled ‘a real gap in the N.H.S.’ and that there was ‘virtually no other way a woman can find out if she is pregnant, except by persuading her doctor to arrange a test, or to pay for one to be done’. The problem, as they saw it, was that Bristol doctors didn’t perform the tests themselves, but instead sent samples to the local Public Health Laboratory or Southmead Hospital. Often, a woman was required to take the sample there herself and, in any case, she would not be informed of the result for several days and, then, was ‘only told indirectly via her doctor.’ Moreover, the Bristol testers had ‘heard many instances of samples getting mislaid and the results getting lost.’^52^

Suspicious samples emerged as a new concern after a series of articles published in the *News of the World* in February and March 1974 alleged that laboratories were providing false positive test results to generate business for abortion clinics. These and other allegations were later reproduced in the book, *Babies for Burning*.^53^ Members of the Bristol group returned from W.A.C.C.’s second annual conference in Nottingham, in March 1975, with ‘some pretty hairy stories from people in PAS who had been given doctored samples by anti-abortionists trying to cast doubts on their reliability and honesty.’^54^ Most women did not produce a sample on site, but brought one along, so to cover themselves from allegations of fraud the Bristol group adopted a policy of ‘stating that the test is 98% accurate, and that if it is a fresh sample in a clean bottle, and not taken too early after missing a period, then we feel reasonably confident about the result.’^55^

## Domesticating a medical technology

**Figure 3.**
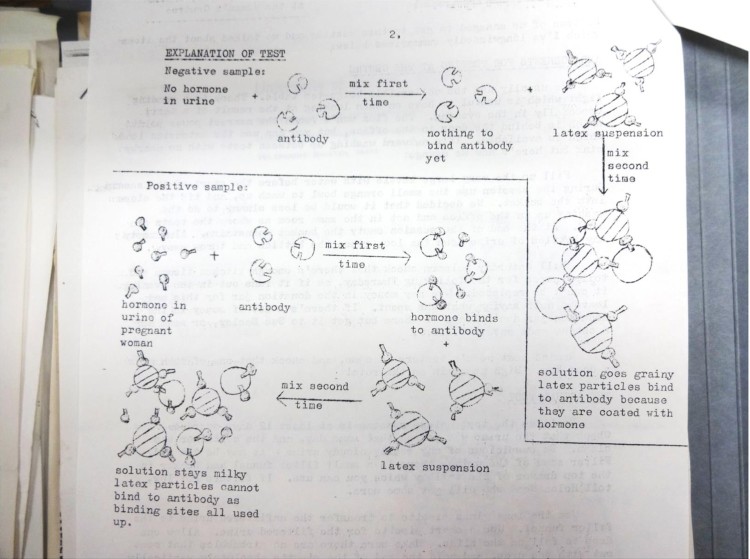
Detail of report on ‘Pregnancy Testers’ Meeting’, Women’s Centre, Bristol, 17 February 1976. Report and diagrams by Helen Seed, typed by Betty Underwood. Jackie West materials on abortion, contraception, and other topics, 1912–1983, University of Bristol Library Special Collections, Bristol (DM2614/2).

As with cervical self-examination and menstrual extraction, feminist pregnancy testing depended on the appropriation and circulation of inexpensive and portable tools and protocols.^56^ The Bristol group took pains to explain the immunological science behind pregnancy testing (Figure 3), but the tester—whether a lay activist, lab technician, pharmacist, or G.P.—did not need to understand how the test worked at the molecular level to become adept at using the kit. C.P.A.G.’s Mary Bernard, for instance, had ‘no scientific knowledge whatsoever’, but rapidly learned the basics of reproductive physiology and ‘could explain to people what the test was about.’^57^ Just as lab technicians had learned to skilfully manipulate test animals, so too did activists learn the basic laboratory skill of mixing reagents with urine and interpreting the results.^58^ On the other hand, many testers were able to draw on prior experience. Lucy King, for instance, had studied biology in Scotland, and a female doctor in the Bristol group trained others to use the kit.^59^ The medical laboratory has often been compared with the kitchen and it is also possible that gendered household skills facilitated the domestication of pregnancy testing, which could be a bit like cooking.^60^

C.P.A.G. obtained Organon’s Pregnosticon Planotest from the Dutch pharmaceutical company’s British laboratories in Morden, Surrey. Organon did not supply ‘members of the public’, so the group approached Sheila Abdullah, a ‘sympathetic’ Liverpool doctor and abortion campaigner they had met at the W.A.C.C. conference, and she agreed to obtain the kit for them.^61^ Planotest simply required the user first to mix one drop of urine with two drops of commercially prepared liquid reagents on a glass slide, and then read the result after two minutes of ‘gentle mixing’. Cambridge testers worked in pairs to confirm one another’s readings and generally became confident in distinguishing between positive and negative results after seeing about six tests each. Good lighting was important to read the test correctly and testees were instructed to bring their urine samples ‘in a clean bottle, well rinsed with water, not detergent, as this may interfere with the reaction.’ Eventually, Abdullah was able to vouch for the group’s reliability and Organon agreed to supply Planotest directly. C.P.A.G. offered to order and sell kits to newly established groups in other parts of the country until they were able to ‘set up a similar arrangement locally.’^62^ By May 1975, they had performed around 600 tests, and knew of only one incorrect result.

**Figure 4.**
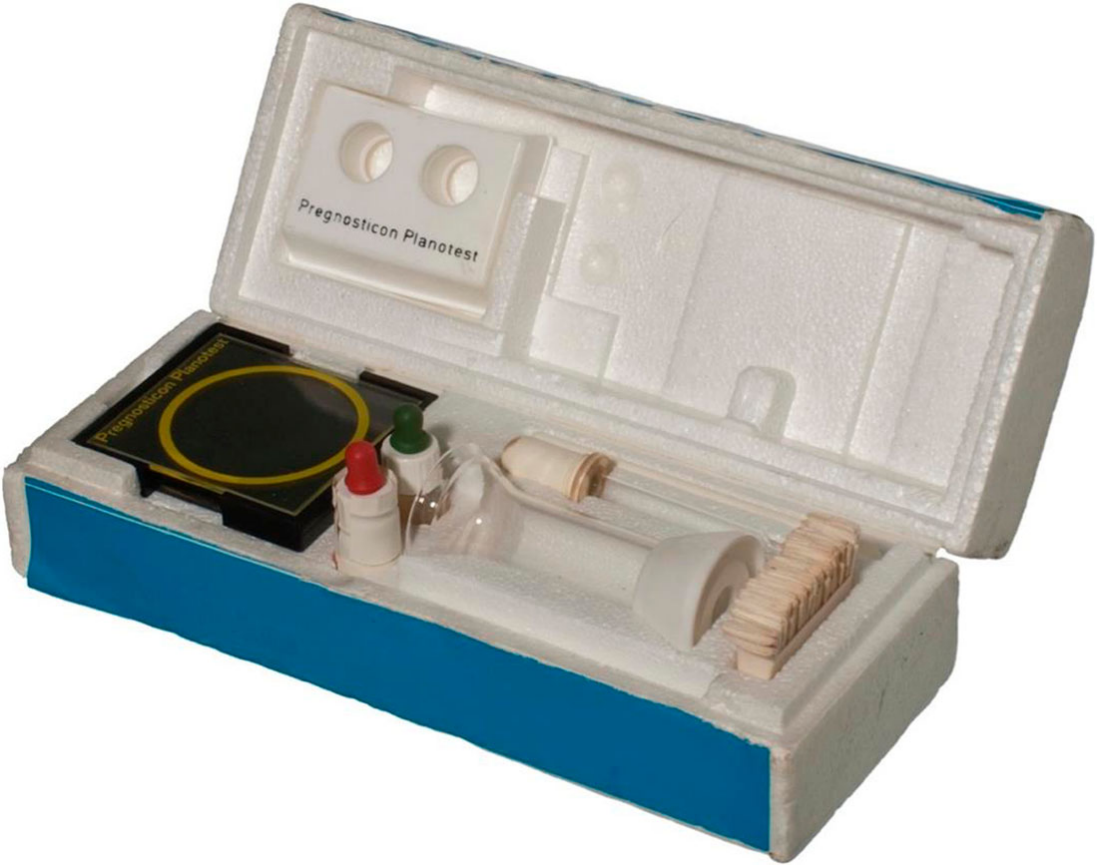
Pregnosticon Planotest, 1967, 19.4 × 7.8 × 5.8 cm, Museum of Contraception and Abortion, Vienna, Inventory number: 2747. Note the red and green topped bottles.

The instructions for Pregnosticon Planotest, preserved along with an example of the test kit at the Museum of Contraception and Abortion in Vienna (Figure 4), read as follows:

Place 1 drop of antiserum within the yellow circle on the slide and add 1 drop of urine with the spare dropper provided. Mix carefully with the white spatula. Shake the latex suspension and add 1 drop to the mixture on the slide. Stir this mixture with the spatula and spread it over the entire surface within the yellow circle entirely. Shake the slide gently for two minutes so that the liquid covers the yellow circle evenly. The test result can then be checked. No agglutination: positive for pregnancy. Agglutination: negative for pregnancy.

Activists familiarised themselves with the jargon of immunochemistry and learned to translate it into plain English. Agglutination, for instance, was likened to ‘ground glass’ and its absence described as ‘milky looking’ in the instructions prepared by a member of the Kingsgate Place Women’s Centre in Northwest London:

Stuff is in fridge. Take an early morning sample of urine. Put 1 drop on clean glass slide in box with dropper. On top of that put 1 drop of solution from green topped bottle to start reaction. Add to that 1 drop from red bottle. Be sure to keep urine and chemicals in circle on glass slide. Move liquid around slowly in circular motion for several minutes. If it remains milky looking, thick and white test is positive (she’s pregnant). If it forms small granules (like ground glass) it’s a negative test. Helps if you hold it up to light or have several people look.^63^

A fridge, which could be found in the majority British households by the early 1970s, was important.^64^ A note from the same centre, dated Thursday 21 November 1974, reads, ‘Fridge broken so pregnancy testing not possible until further notice’. (Planotest stuff must be kept at 4°.)’^65^ The Bristol group also had refrigeration trouble. In November 1975, they were ‘still looking for a fridge so that we can keep the pregnancy testing kit at the women’s centre instead of passing it from hand to hand (or rather, fridge to fridge) as at present.’^66^

Privacy, or lack thereof, was another issue. Who knew the result? Just the doctor? The receptionist too?^67^ A 1977 B.P.A.S. report observed that ‘requesting a test within the hearing of a waiting room full of people is not conducive with the ideal of what should be a confidential health service.’^68^ Women’s groups did what they could, but the domestic space of pregnancy testing was in short supply. Malos’s basement doubled as a shelter for homeless women, including, on one occasion, ‘a young woman […] who was very heavily pregnant, kind of lying on the bed while women were coming in for pregnancy tests.’ This prompted the Bristol group to develop ‘a rule that women couldn’t stay […] beyond Friday evening, because pregnancy testing happened on Saturday morning.’^69^ Potentially upsetting intrusions, by women who visited the centre to ‘buy books, chat or meet friends,’ had also to be guarded against.^70^

## The ‘many faces’ of pregnancy testing

Why did women in the liberation movement and beyond perceive pregnancy testing as important? For some it was a practical form of involvement in a movement that also tackled large and seemingly intractable problems. As Ruth Wallsgrove later recalled in *Spare Rib*:

Some of us choose the broader, apparently grander visions of a nuclear-free world or total revolution; some choose to organise a pregnancy-testing evening around the corner, knowing for sure that we’ll help a few women, giving them a chance to decide what they will do with their lives.^71^

For others it was a way to bide time while doing something useful. For instance, when the recently divorced Sally Harrison first came to Bristol from America, she decided to take up pregnancy testing while looking around for other activities in which to involve herself because it was a ‘really constructive thing to do’ and ‘easily learned’.^72^ For Betty Underwood of the Bristol W.A.C.C., pregnancy testing was ‘an important link in the chain of facilities which are needed for women to have the right to choose when, and if, they are to have children.’^73^ And for Sue Jones of Bolton, pregnancy testing was ‘one of the important things that we did, because it provided a free service to women, by women who were in the same boats themselves.’^74^

Non-medical pregnancy testing was needed, a C.P.A.G. manifesto argued in 1976, because ‘the NHS works through doctors, GPs whose women patients are often quite justly reluctant to visit them unless they are either ill or desperate.’^75^ While keen to ‘demonstrate some of the inadequacies of the NHS’, the group also tried ‘to put pressure on the NHS to give a better and more human service’ and did not ‘want merely to do their work for them.’^76^ A mission statement by members of the Bristol group explained they had ‘started the free pregnancy testing because as women we know how difficult it can be if our doctors are unsympathetic.’^77^ Other lines of evidence corroborate the perception that some, though not all doctors were indeed out of touch with women’s expectations. To give an example, in 1979 a Norwich G.P. complained in a letter to his M.P.:

The other day I was asked by a patient to supply a bottle and form so that she could have a pregnancy test organised at the pathology laboratory. It appeared that her reason for doing so was idle curiosity, there being no real medical reason for it. On my explaining that social pregnancy testing was not necessarily a part of the National Health Service she became most annoyed and informed me that all her other doctors had done it without question before.^78^

At least one woman chose to have her urine tested at the Brighton women’s centre because her G.P. ‘laughed at her.’^79^

*Ad hoc* counselling, meanwhile, emerged as a significant feature of grassroots pregnancy testing around the country, but the same social advantages that facilitated educated activists’ acquisition of the ‘easy’ laboratory skill could also impede communication across class boundaries. Cambridge testers, for instance, valued counselling as ‘one of the most important, but also the most difficult of the things’ they were trying to do. They felt that it was ‘much simpler to learn to do the pregnancy test than […] to learn how to get through to some of the very varied women who come to us.’ In addition to explaining the test, they asked each woman whether or not she had been using contraception and, if so, whether she wanted to change method and ‘whether she knows what methods are available and where to get fixed up with them.’ Some testees, evidently, had ‘never before talked to anyone about contraception,’ and Cambridge testers sometimes found themselves ‘discussing very basic anatomy and physiology.’^80^

**Figure 5.**
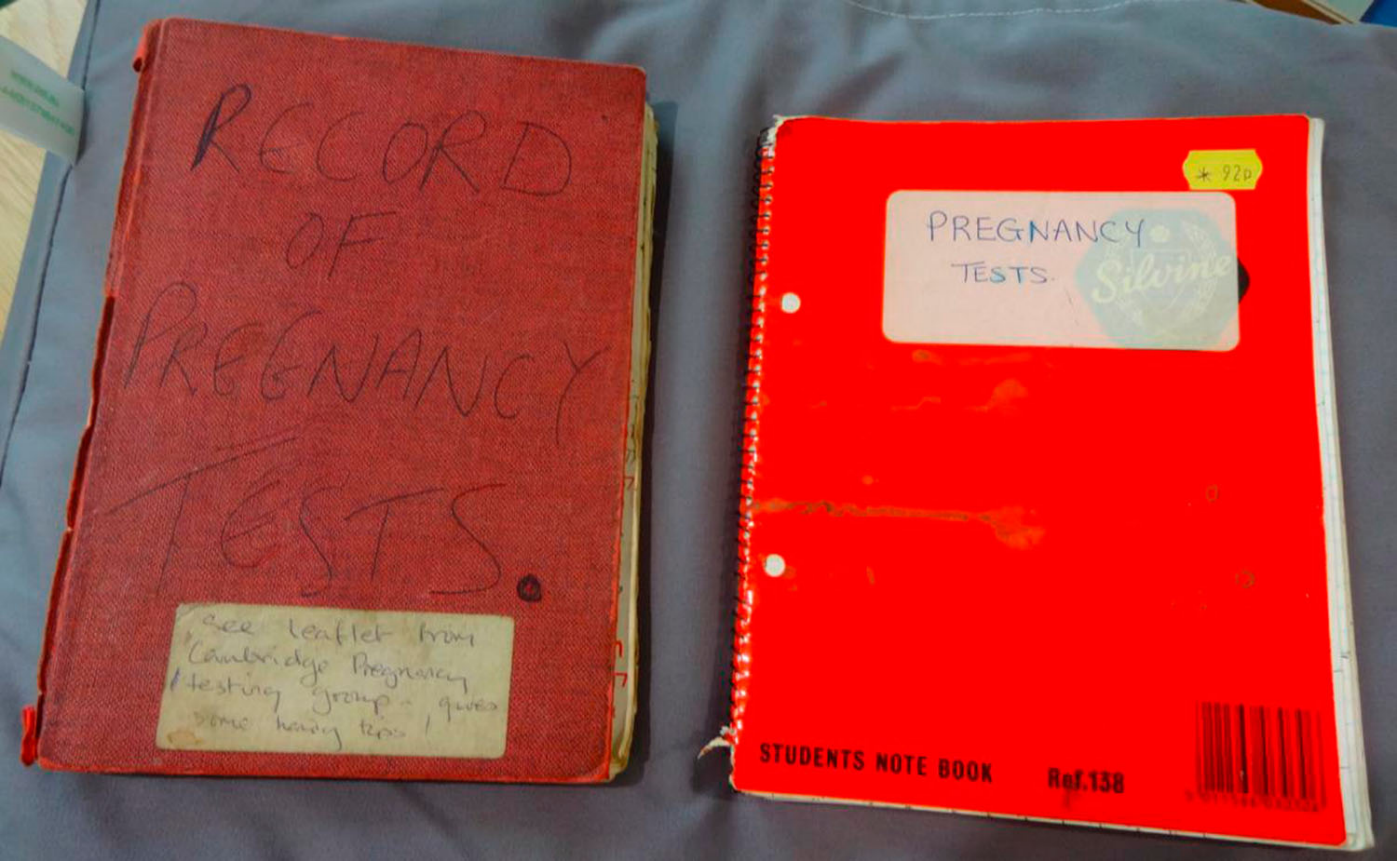
Red ledger and student note book used to log pregnancy tests in the 1970s and 1990s, respectively. Records of the Brighton Women’s Centre, Women’s Library @ LSE, London (5BWC/survey/G).

Records kept by the Brighton service reveal that while some testees did without any form of contraception or were relying on the rhythm method, others had used the sheath, cervical cap, I.U.D., and oral contraceptive pill (Figure 5). The surprisingly high number of women taking the pill who came in for pregnancy testing demonstrated, according to the B.P.A.S. report, ‘the confusion surrounding the pill and also […] the fact that many women are not as happy on the pill as some doctors would have us believe.’^81^

While some members of the Bristol group ‘felt strongly in favour of asking women what contraceptives, if any, they were using’, others ‘felt that sometimes this would be difficult and a bit intrusive.’ They also worried that raising the subject of contraception assumed that a woman was ‘worried about being pregnant’, and knew from experience that ‘at least two out of every five’ women were ‘really pleased to find that they are.’ Eventually, the group decided to ‘play it by ear’ and to ‘try to give women the opportunity to talk about contraception in case they want to, but feel embarrassed about opening the subject.’^82^ Revisiting the issue later on, they reiterated the point that just because they were ‘providing a service,’ it did not give them the ‘right to ask extra questions’ that testees might perceive as ‘unnecessary and prying.’ As an indirect alternative, testers could ‘always invite women to help themselves to any of our leaflets on their way out.’ They also stressed that it was ‘important not to baldly ask women if they want to be pregnant or want an abortion.’ Instead, testers were supposed to say ‘if the test is positive or negative, not “you are pregnant” or “you are not pregnant”. Whatever the result the woman ought probably to see a doctor.’^83^

Sue Jones later recalled that Bolton testers ‘didn’t give advice’ or ‘tell people what to do,’ but rather:

used to give people options of where they could go, you know, or who they could see, and just talk really for who came to see us, about whether the pregnancy test was positive or negative, if they wanted to stay and talk about it, we’d do that.^84^

As with Lucy King in Cambridge, Jones later went on to a professional career in counselling, for which her early experience in pregnancy testing held her in good stead. But for others, impromptu counselling without proper training could take its toll.

One of the most detailed and moving accounts is that of feminist writer Michèle Roberts, who volunteered with a ‘small team of testers […] in a shabby basement, unobtrusively entered down a steep flight of steps,’ at the London P.A.S.^85^ Roberts had no training in counselling and her instructions were to refer her clients to their G.P.s or else to the trained counsellors upstairs. As she later recalled in *Paper Houses*, her memoirs of the 1970s:

Some women, receiving their test results, expressed delight, because they wanted to get pregnant, and rushed away. Some were grateful for the contraceptive information we gave them. Others were devastated to be told they were pregnant, burst into tears and then poured out their unhappy stories. Of course I listened. I could not possibly show women to the door while they sat in front of me weeping. They recounted tales of male doctors’ and boyfriends’ callousness, indifference, brutality. I did not know so much suffering existed. I did not know how to listen in a detached way: I took the women’s grief and anger into myself and carried it home with me […] One morning I just collapsed into tears. I wept into the urine sample I was testing and ruined it and the woman who’d given the sample had to go and supply another one and was understandably annoyed. I was told to take the day off. I went home but didn’t know how to cope. I just went on crying.^86^

Though Roberts’s account understandably concentrates on the grief and despair of unhappily pregnant women, it also helpfully draws attention to the delight experienced by some happily pregnant testees. While direct evidence is scarce, records have survived that permit a partial reconstruction of the testee’s perspective, albeit a mediated one. For instance, the red ledgers kept by the Brighton group record the highly variable reactions of testees: ‘Ah well—try harder!’; ‘Thank God for that!’; ‘Relieved’; ‘Bit disappointed’; ‘Going to doctor—still unsure!’; ‘Sad but wants to keep it’; ‘Pleased’; ‘Very pleased’; ‘Very very pleased’; ‘Oh gosh’; ‘Stunned’; ‘Shocked’; ‘Wonderful’; ‘Don’t mind’; ‘Going to have an abortion’; ‘Fantastic’; ‘Great!’^87^

Aggregated and analysed data confirms that pregnancy testing, if nothing else, was a variable experience. Of the sixty-two positive results obtained by C.P.A.G. in a single year, twenty women ‘wanted to be pregnant,’ thirty ‘did not’ and the rest either ‘weren’t sure’ or didn’t say.^88^ An analysis of 304 tests performed by the Bristol service found that, while 53% of testees were pleased with a negative result and 43% disappointed with a positive, 37% were pleased with a positive and 13% disappointed with a negative. No older women wanted to be pregnant and sufficiently many were worried about menopause for the group to produce an informational leaflet on the ‘Changes in Life’. As expected, young women and girls frequently ‘wanted reassurance after “taking a chance,”’ or else doubted the effectiveness of the contraceptive they were using. But a substantial group of women in their mid to late twenties were ‘keen to start or add to their families’ and ‘really pleased to get a positive result.’^89^ As the title of the B.P.A.S. report put it, pregnancy testing had ‘many faces’.

B.P.A.S. began in Birmingham in 1968 as an abortion provider, but a decade later its Sheffield branch was also providing referrals for infertility treatment, albeit to a ‘small minority of women with, apparently, long histories of trying [to conceive] and who were clearly upset by a negative result.’ Just over one in four (27.4%) of all women known to have had their first pregnancy test with the Sheffield B.P.A.S. were ‘anxious to be pregnant.’^90^ In Bristol, Ellen Malos had expected the pregnancy testing service to be used by women ‘who didn’t want to be pregnant’ and was surprised that many testees ‘were women who were quite happy to be pregnant but just wanted to know’ or were ‘older women who were having menopause babies and didn’t quite know what they thought about it’.^91^ And in Bolton, Sue Jones remembered that, in the days ‘before we heard about test tube babies or infertility or anything,’ the service attracted ‘a number of married women coming who couldn’t get pregnant’ and who used it ‘for as they would call it now “fertility problems.”’ She further reflected that this:

was an indication of something that was really going to grow into a big … well, it is a big industry now isn’t it? All happening all the way back in the early 1970s. So there was a need for it, well, there had obviously been a need for it before, but there was a real need beginning for it then.^92^

At the level of national campaigning, the women’s liberation movement did not organise anything on the scale of W.A.C.C. for menopause or infertility. Bristol’s Angela Roddaway, who joined the movement when she was in her fifties, struggled for years to start a discussion group on menopause,^93^ and Naomi Pfeffer’s attempts to set up workshops on infertility at women’s health conferences in the early 1980s ‘met with no response’.^94^ At the level of grassroots activism, however, feminists such as Sue Jones, Ellen Malos and Michèle Roberts were confronted by a spectrum of women seeking tests for a variety of reasons. B.P.A.S., P.A.S. and W.A.C.C. initially set up pregnancy testing services to facilitate access to contraception and abortion. But testees, including women struggling with fertility problems, menopausal women and young, happily pregnant women challenged the assumptions and expectations of volunteer testers, some of whom later came to see themselves as having been in the vanguard of access to information about infertility and menopause.

## Winding down

Many women, and a few men, continued to organise local pregnancy testing services in the late 1970s. A mixed group including medical students started the Oxford Pregnancy & Abortion Support Group in 1976 to provide an alternative to LIFE, which was ‘widely publicizing its covertly anti-abortion pregnancy testing/counselling services.’ They offered free pregnancy testing, by appointment only, on Tuesday evenings and Saturday mornings (donations ‘gratefully received’ to offset the 30p cost of each test), and also ran a counselling service. In February 1976 the group was ‘pushing for both these services to be provided by the NHS.’^95^ The Archway women’s health group in North London met for pregnancy testing and, in 1977, planned practical workshops on health, electricity, massage, photography, plumbing and bicycles. In Camden, free pregnancy testing was offered alongside feminist therapy, health groups, study groups, yoga and karate.^96^ A Norwich collective, based in 1978 in a room in the Community Arts Centre, concentrated on ‘free pregnancy testing and advice/support to women.’^97^ And the women’s group in Newham, East London, launched a service in 1978, to ‘enormous’ demand.^98^

Women attending the seventh national Women’s Liberation Conference in Newcastle in April 1977 discussed pregnancy testing in relation to ‘the problem of how much to create alternative structures and how much to put pressure on the NHS to provide what women need.’^99^ In July 1977 the National Pregnancy Testers’ Conference at the Friends Meeting House in Bristol debated the extent to which women’s groups aimed ‘to provide an exemplary service and pressurise the NHS’ or considered ‘a woman-run pregnancy testing service as something valuable which can’t be provided by paid professional workers—both for ourselves, as lay women developing a skill, and for women who want a test and can see one being done without mystique.’^100^ At least two further national pregnancy testers’ conferences were held, in May 1978, at Newnham College, Cambridge, and in June 1981, at the Queen’s Walk Community Centre in Nottingham.^101^

Feminist guides such as *The Woman’s Directory* and *The Women’s Health Handbook*, both published in 1976 by Virago, advertised the addresses of local groups.^102^ A revised edition of the latter from 1978 discouraged the use of ‘difficult to read’ home tests, which ‘may have been stored incorrectly and deteriorated when not refrigerated, or the reagents may be out of date.’^103^ The first British edition of *Our Bodies, Ourselves*, published by Penguin in 1978, recommended ‘WL groups’ over commercial agencies, since the former were ‘obviously committed to providing accurate results. Not all commercial agencies can be so relied on.’^104^ And Virago’s *Talking to Your Doctor*, also published in 1978, ‘particularly’ recommended clinics ‘run by women’s groups,’ because they did pregnancy testing:

for nothing and tell you the results immediately, whereas a doctor will send away your urine sample and this may mean waiting up to 10 days for the result—too long if you think you may want an abortion should the result be positive.^105^

For members of the Bristol group, pregnancy testing was an ‘important’ activity, and so they were ‘unhappy’ when the number of volunteers began to drop off. The service ‘only managed to keep going’ because of ‘a few dedicated people’ and, while the group was able to recruit new testers and to ‘resurrect’ a few old ones, filling the rota became a perennial problem.^106^ In November 1976, for example, the rota was ‘still very empty’ and the group reminded readers of the *Bristol Women’s Liberation Newsletter* that they ‘always need more testers; the test is very simple and it’s easy to learn.’^107^ Finally, in December 1979, about six years after it had been set up, the labour shortage was precipitating a ‘crisis’. The centre had ‘not yet had to cancel any sessions,’ but it had come close ‘on many occasions.’ If the service became unreliable, an open letter warned, the group would be ‘just as guilty as the NHS for messing women around.’ Pregnancy testing, the letter reasserted, ‘is an important function of the Women’s Centre.’ It was ‘easy to learn, quick, reliable, and done in a supportive environment that avoids the waiting and bureaucratic nonsense required by the NHS’ and it had ‘helped thousands of women who would otherwise not have made any contact with the Women’s Centre.’ For testers, it took only a couple of hours every month or so and could be ‘a very rewarding way to spend a Saturday morning or Thursday evening.’^108^

**Figure 6.**
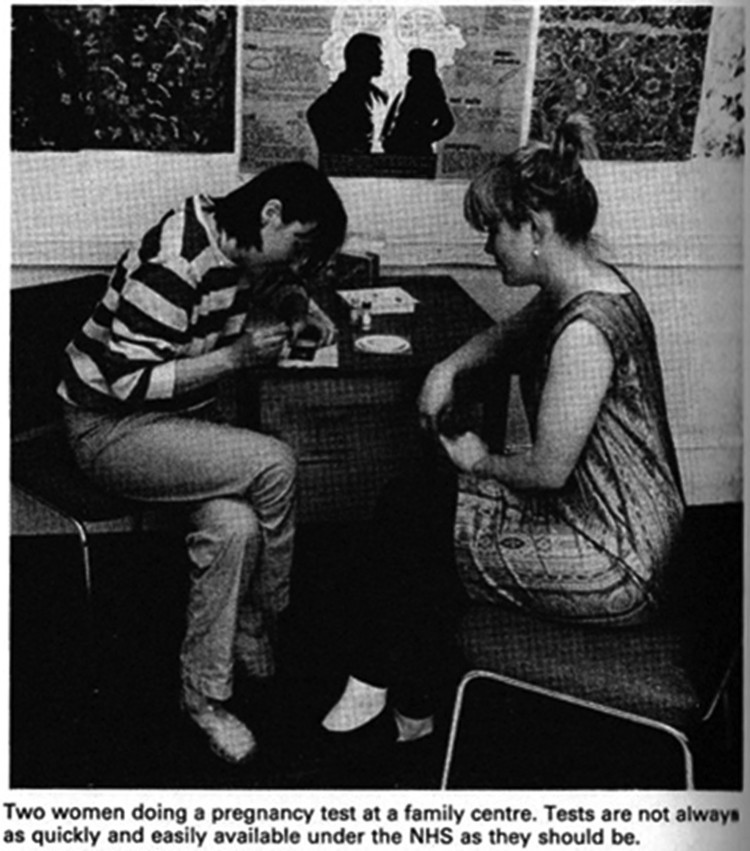
Image of pregnancy testing as women helping women in Nick Davidson & Jill Rakusen (1982) *Out of our Hands* (London: Pan), p. 60. Photo credit: Gina Glover (Photo Co-op). The test kit appears to be Pregnosticon. Reproduced by kind permission of the Syndics of Cambridge University Library.

**Figure 7.**
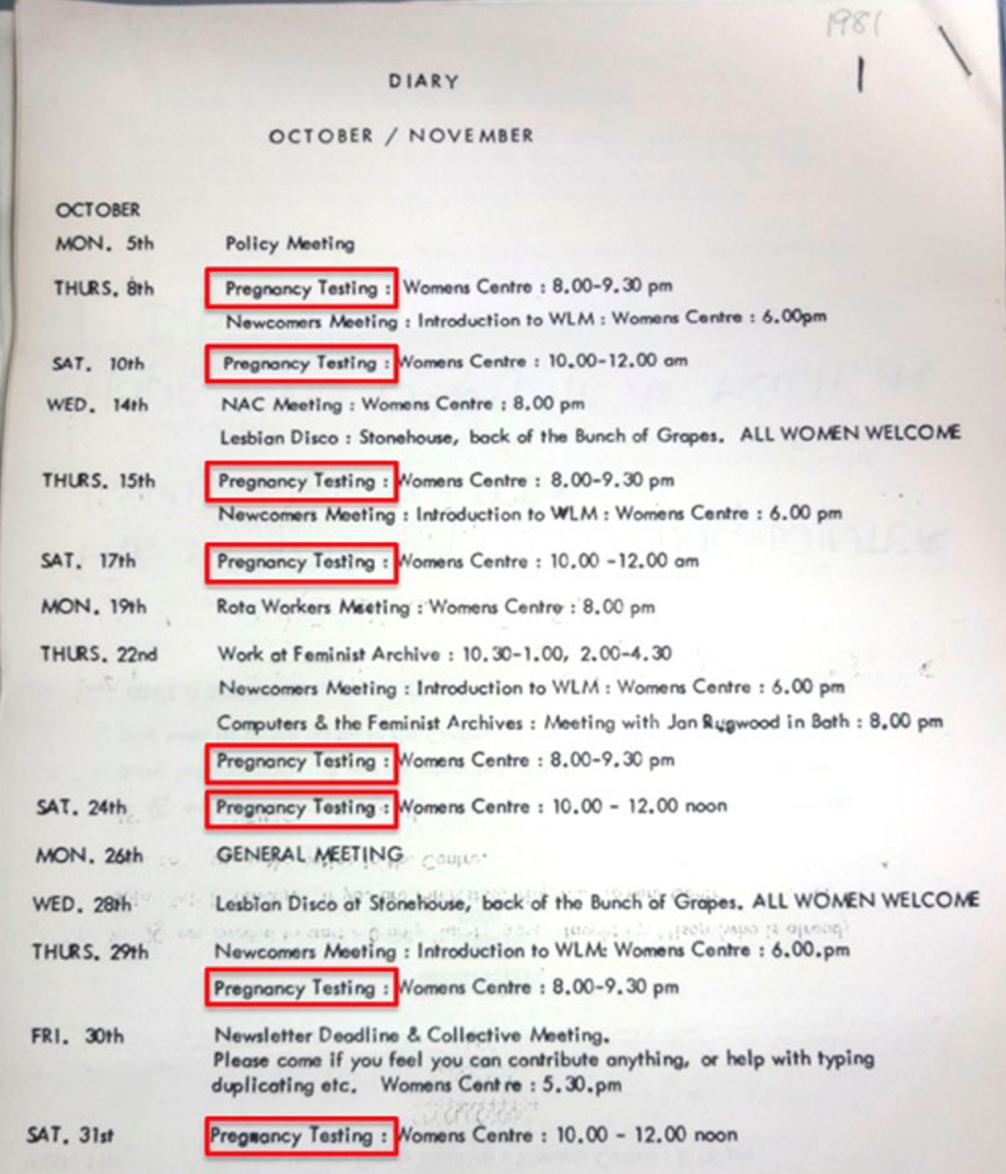
*Bristol Women’s Liberation Newsletter*, October 1981, Ellen Malos Archive, Feminist Archive South, University of Bristol Library Special Collections, Bristol (DM2123/8/115).

Despite shrinking donations and rota problems, feminist pregnancy testing persisted into the 1980s, well beyond the final, acrimonious national conference of the women’s liberation movement in Birmingham in 1978 (Figure 6).^109^ Activist Elizabeth Bird later remembered using the Bristol service ‘at the Women’s Centre when she found out she was pregnant with her son (on International Women’s Day!) in 1981.’^110^ Pregnancy testing accounted for nearly 40% (8 out of 21) of all activities scheduled by the centre in October 1981, alongside N.A.C. meetings, lesbian discos and computer work at the Feminist Archive (Figure 7). The service was still going in 1983, when the Bristol *Newsletter* ends.^111^

The pregnancy testing service at a women’s centre in Paignton, Devon, was still going in 1982, though struggling to cover costs.^112^ Free pregnancy testing carried on in the London Borough of Brent even after the local women’s centre closed in 1992.^113^ The Brighton group, which originally ran from 1975 to 1981, was revived between 1991 and 1996.^114^ And some ninety people had volunteered for C.P.A.G. by the time the last urine sample was tested in September 1993.^115^ By then, women’s health centres had given way to a network of feminist-run ‘well women’s clinics’ that attempted, with mixed results, to provide an alternative model within a restructured N.H.S.^116^ Doctors too had changed; a younger generation of G.P.s and gynaecologists were more understanding of women’s needs than their paternalistic predecessors had been.^117^ Meanwhile, the commercial rise of Clearblue and other more streamlined and aggressively marketed products decisively put an end to drop-in services and brought self-testing from the margins into the mainstream, creating a new normal for a younger generation of women.^118^ Somewhere along the way, the plastic speculum and menstrual extraction kit, but not the pregnancy test, came to be celebrated as iconic relics of ‘second-wave’ feminism.^119^ Today, the utter commonplaceness and slick commercialism of pregnancy testing conceal its radical past.

## A feminist technology?

This article has recovered one of the central activities of the women’s liberation movement: free or at-cost, anonymous on-the-spot pregnancy testing usually combined with counselling, information about contraception and referrals for antenatal care or abortion. Although initially linked to W.A.C.C., feminist pregnancy testing was not clearly delimited by the movement’s official demands. Rather, its remit extended to include infertility and menopause as well. As with the networks of birth-control and family planning clinics that went before, the pregnancy testing services set up in the 1970s also served women who were trying to conceive.^120^ Nor were such services obviously part of a broader women’s health movement, as it was in America. Rather, they were offered alongside a jumble of activities—from yoga and karate to bicycle repair and women’s studies. British feminists framed pregnancy testing as a more efficient and sympathetic alternative to the inadequate N.H.S. as they pushed for reform. They appropriated a medical technology, bringing it into the domestic sphere and endowing it with the politics of the movement.

Many women’s groups ‘made serious attempts to reach local working class women based on their own particular needs.’^121^ Pregnancy testing was one of these needs and, for the predominantly white middle-class activists, it provided a means of reaching a diversity of women, including those who had never before talked to anyone about contraception, anatomy or reproduction, and who would otherwise not have made contact with a women’s centre.^122^ In this regard, it is helpful to think about feminist pregnancy testing in relation to comparatively well-studied instances of health-related activism. The Black Panther Party, for instance, used Sickledex, an inexpensive, portable diagnostic test for sickle cell anaemia to not only raise awareness about the genetic disease, but also recruit members and garner support for related causes.^123^ A.I.D.S. activists, often educated white middle-class men, acquired the language of immunology and learned to translate it into the vernacular, much as feminists had done before.^124^ And the birth-control clinics set up by Marie Stopes and others, including Lella Secor Florence in Cambridge, confronted similar challenges in reaching the desired clientele, including rural and working-class women.^125^

The well-documented demand for feminist pregnancy testing combined with the paucity of historical evidence for self-testing may give the impression that ‘no self-administered kits were available’ in the 1970s.^126^ Nevertheless, it is important to remember that Predictor and other home tests were in fact stocked by many British pharmacies, though not Boots, from 1971. This does not mean, however, that self-testing was the norm. On the contrary, waiting on one or the other side of the bathroom door for the result of a self-administered test only started to become a commonplace around 1990. Until then, self-testing coexisted with a range of medical and non-medical services that offered pregnancy testing to the whole spectrum of women. Predictor, which still had to be purchased in public and somehow disposed of, did not guarantee privacy and many women opted for anonymous drop-in services, including those provided free of charge by feminists.^127^ What feminist pregnancy testing lacked in convenience, it more than made up for with supportive counselling, reliable information and considered referrals. Perhaps contrary to present-day expectations, the most subversive aspect of pregnancy testing in the 1970s was not privacy, but counselling—a sympathetic ear and shoulder to cry on. As with the interwar birth-control clinics, compassionate ‘woman-to-woman’ care distinguished feminist pregnancy testing from competing alternatives.^128^ In the hands of lay activists the pregnancy test kit became, at least for a time, a feminist technology.^129^

## Notes

1 Janine Perry’s interview with Sue Jones, 11 January 2009, Feminist Archives North, Special Collections, Leeds University Library, Leeds (FAN/BWLG), original italics.

2 Sue Bruley (2016) Women’s Liberation at the Grass Roots: a view from some English towns, *c*.1968–1990, *Women’s History Review*, 25, pp. 723–740.

3 Lesley Doyal (1983) Women, Health and Sexual Division of Labour: a case study of the women’s health movement in Britain, *Critical Social Policy*, 7, pp. 21–33, p. 21. For the American movement: Sandra Morgen (2002) *Into Our Own Hands: the women’s health movement in the United States, 1969–1990* (New Brunswick NJ: Rutgers University Press); Wendy Kline (2010) *Bodies of Knowledge: sexuality, reproduction, and women’s health in the second wave* (Chicago: University of Chicago Press); Michelle Murphy (2012) *Seizing the Means of Reproduction: entanglements of feminism, health, and technoscience* (Durham NC: Duke University Press); Jennifer Nelson (2015) *More than Medicine: a history of the feminist women’s health movement* (New York: New York University Press).

4 Sue O’Sullivan (1987) *Women’s Health: a* Spare Rib *reader* (London: Pandora); Ellen Annandale (2009) *Women’s Health and Social Change* (London: Routledge), pp. 42–45; Natalie Thomlinson (2016) *Race, Ethnicity and the Women’s Movement in England, 1968–1993* (New York: Palgrave), pp. 61–62.

5 Lesley Doyal (2006) Sex, Gender and Medicine: the case of the N.H.S., in David Kelleher, Jonathan Gabe & Gareth Williams (Eds) *Challenging Medicine*, 2nd edn (Abingdon: Routledge), p. 151.

6 Doyal, ‘Women, Health and Sexual Division of Labour’, pp. 23, 31.

7 Suzanne Staggenborg (1991) *The Pro-choice Movement: organization and activism in the abortion conflict* (New York: Oxford University Press), pp. 49–50. See also Sarah A. Leavitt (2006) ‘A Private Little Revolution’: the home pregnancy test in American culture, *Bulletin of the History of Medicine*, 80, pp. 317–345, p. 328.

8 Deborah Cohen (1993) Private Lives in Public Spaces: Marie Stopes, the Mothers’ Clinics and the practice of contraception, *History Workshop Journal*, 35, pp. 95–116.

9 Audrey Leathard (1980) *The Fight for Family Planning: development of family planning service in Britain, 1921–74* (London: Macmillan); Clare Debenham (2014) *Birth Control and the Rights of Women: post-suffrage feminism in the early twentieth century* (London: I.B. Tauris).

10 B.P.A.S. became the *British* Pregnancy Advisory Service in the mid 1980s and merged with P.A.S. in the late 1990s: David Paintin (2015) *Abortion Law Reform in Britain 1964–2003: a personal account by David Paintin* (Stratford-upon-Avon: B.P.A.S.), pp. 66–74. On Brook: *Wellcome Witness Seminar: 50 years of Brook on Friday 6th February 2015*, http://film.wellcome.ac.uk:15151/0056-0000-8081-0000-0-0000-0000-0.pdf, transcript accessed 12 September 2016.

11 Margaretta Jolly, Polly Russell & Rachel Cohen (2012) Sisterhood and After: individualism, ethics and an oral history of the women’s liberation movement, *Social Movement Studies*, 11, pp. 211–226.

12 More recently, women-led communities have similarly appropriated the ‘abortion pill’, mifepristone (RU486) to demystify abortion and decentre the doctor’s role: Sally Sheldon (2014) The Medical Framework and Early Medical Abortion in the UK: how can a state control swallowing? In Rebecca J. Cook, Joanna N. Erdman & Bernard M. Dickens (Eds) *Abortion Law in Transnational Perspective: cases and controversies* (Philadelphia: University of Pennsylvania Press), pp. 189–209.

13 See Laura Kaplan (1995) *The Story of Jane: the legendary underground abortion service* (Chicago: University of Chicago Press), p. 172; Johanna Schoen (2015) *Abortion After Roe* (Chapel Hill: University of North Carolina Press), pp. 46–47.

14 For accounts of self-diagnosis in the 1970s: Brigitte Jordan (1977) The Self-Diagnosis of Early Pregnancy: an investigation of lay competence, *Medical Anthropology*, 2, pp. 1–38; Ann Oakley (1979) *Becoming a Mother* (Oxford: Martin Robertson), pp. 27–28.

15 Marie C. Stopes, *Mother England: a contemporary history self-written by those who have had no historian* (London: Bale), pp. 29–31; Mark Marshall (1948) The Kyesteine Pellicle: an early biological test for pregnancy, *Bulletin of the History of Medicine*, 22, pp. 178–195. For the earlier history: Michael Stolberg (2015) *Uroscopy in Early Modern Europe* (Farnham: Ashgate), pp. 82–91.

16 Jesse Olszynko-Gryn (2014) *Pregnancy Testing in Britain, c.1900–67: laboratories, animals and demand from doctors, patients and consumers* (Ph.D. thesis, University of Cambridge).

17 Olszynko-Gryn (2013) When Pregnancy Tests Were Toads: the *Xenopus* test in the early N.H.S., *Wellcome History*, 51, pp. 1–3; Olszynko-Gryn (2014) The Demand for Pregnancy Testing: the Aschheim-Zondek reaction, diagnostic versatility, and laboratory services in 1930s Britain, *Studies in History and Philosophy of Biological and Biomedical Sciences*, 47, pp. 233–247.

18 W. W. Holland & T. P. Whitehead (1973) Value of New Laboratory Tests in Diagnosis and Treatment, *Lancet*, 304, pp. 391–394; Martin Gorsky (2013) ‘Searching for the People in Charge’: appraising the 1983 Griffiths N.H.S. management inquiry, *Medical History*, 57, pp. 87–107, p. 89.

19 See Salim Al-Gailani (2014) Making Birth Defects ‘Preventable’: pre-conceptional vitamin supplements and the politics of risk reduction, *Studies in History and Philosophy of Biological and Biomedical Science*s, 47, pp. 278–289.

20 Ann Oakley (1984) *The Captured Womb: a history of the medical care of pregnant women* (New York: Blackwell), p. 230.

21 Olszynko-Gryn, *Pregnancy Testing in Britain*, pp. 249–250.

22 For the relevant market research from 1971: A. B. Giles to Helene Grahame, 9 November 1971, Home Pregnancy Test, Helene Grahame Papers, Wellcome Library, London (PPGRA/B/4). For pregnancy testing as a ‘thoroughly unmentionable subject’ in the 1930s: Margaret Deacon’s interview with Professor Hugh Donald, Edinburgh University Science Studies Unit, 1969–1971, Institute of Animal Genetics, British Library Sound Archive, London (C1271/04/01).

23 Stanley Chapman (2013) The Sexual Revolution, *Southwell Folio*, 9, pp. 32–33.

24 Consumers’ Association (1974) *Sex with Health: the ‘Which?’ guide to contraception, abortion and sex-related diseases* (London: Consumers’ Association), pp. 53–54.

25 Delia Davin, ‘Using commercial pregnancy test in the 1960s’, letter to author, 19 January 2014.

26 I used the National Archives currency converter: http://www.nationalarchives.gov.uk/currency/. Many G.P.s also prescribed, on the N.H.S., Schering’s ‘Primodos,’ a ‘hormonal pregnancy test’ in tablet form that was less expensive and faster than ordering a urine test. The drug, which worked by inducing menstruation in non-pregnant women (a ‘negative’ result), was taken off the market in 1978 amidst concerns that it caused a variety of birth defects: Jesse Olszynko-Gryn, ‘Primodos Was a Revolutionary Oral Pregnancy Test: But Was It Safe?’ *The Guardian*, 13 October 2016, https://www.theguardian.com/science/the-h-word/2016/oct/13/primodos-was-a-revolutionary-oral-pregnancy-test-but-was-it-safe, accessed 6 July 2017.

27 Joni Lovenduski & Vicky Randall, *Contemporary Feminist Politics: women and power in Britain* (Oxford: Oxford University Press), p. 222. On the little-studied anti-abortion movement: Alan Clarke (1987) Moral Reform and the Anti-Abortion Movement, *Sociological Review*, 35, pp. 123–149.

28 Ibid. p. 220.

29 Ashley Wivel (1998) Abortion Policy and the Politics on the Lane Committee of Enquiry, 1971–74, *Social History of Medicine*, 11, pp. 109–135, p. 109.

30 Lane Committee (1974) *Report of the Committee on the Working of the Abortion Act*, vol. I (London: H.M.S.O.), p. 489.

31 Ibid. p. 494.

32 Ibid. p. 499.

33 ‘Pregnancy kits ban urged’, *The Times*, 22 February 1972, p. 4; ‘Women call for ban on birth-test kits’, *Daily Mirror*, 22 February 1972, p. 3.

34 Dorothy McBride Stetson (2001) Women’s Movements’ Defence of Legal Abortion in Great Britain, in Dorothy McBride Stetson (Ed.) *Abortion Politics, Women’s Movements, and the Democratic State: a comparative study of state feminism* (Oxford: Oxford University Press), p. 140.

35 David Bouchier (1983) *The Feminist Challenge: the movement for women’s liberation in Britain and the United States* (London: Macmillan), p. 113; Lesley Hoggart (2003) *Feminist Campaigns for Birth Control and Abortion Rights in Britain* (Lewiston: Edwin Mellon), p. 162.

36 W.A.C.C. (*c*.1972) Women and Abortion: the Women’s Abortion and Contraception Campaign’s evidence to the Lane Commission (London: W.A.C.C.).

37 Ann Oakley (1984) *Taking it Like a Woman* (London: Cape), pp. 77–78.

38 Sheila Rowbotham (1989) *The Past is Before Us: feminism in action since the 1960s* (Harmondsworth: Penguin), p. 65; Elizabeth Bird (2003) Women’s Studies and the Women’s Movement in Britain: origins and evolution, 1970–2000, *Women’s History Review*, 12, pp. 263–288, p. 266; Bridget Lockyer (2013) An Irregular Period? Participation in the Bradford women’s liberation movement, *Women’s History Review*, 22, pp. 643–665, p. 647.

39 Rowbotham, *The Past is Before Us*, p. 75.

40 The Lane Abortion Report: a feminist view, *Spare Rib*, 24, June 1974, p. 22.

41 Bruley, ‘Women’s Liberation’, p. 728.

42 Author’s interview with Mary Bernard, Jenny Corrigall and Lucy King, Cambridge, 13 May 2016 (hereafter ‘Author’s interview’).

43 C.P.A.G. (May 1975) How We Set Up a Pregnancy Testing Group (Cambridge: C.P.A.G.), Pregnancy testing correspondence and papers, Cambridge Women’s Liberation Group Archive, Girton College Archive, Cambridge (GBR/0271/GCIP CWLA). Other centres relegated men to a waiting area, which could be a source of annoyance: Sue Bruley, email to author, 2 June 2016. For men’s involvement in women’s liberation more generally: Nicholas Owen (2012) Men and the 1970s Women’s Liberation Movement, *Historical Journal*, 56, pp. 801–826; Lucy Delap (2017) Uneasy Solidarity: the British men’s movement and feminism, in Kristina Schultz (Ed.) *The Women's Liberation Movement: Impacts and Outcomes* (New York: Berghahn), pp. 214–236.

44 Author’s interview.

45 C.P.A.G., ‘How We Set Up’.

46 Ibid.

47 Ibid.

48 ‘Talking with Women in Bristol’, *Spare Rib*, 102, January 1981, pp. 6–8, 34–35, p. 6; Viv Honeybourne & Ilona Singer (2003) *Personal Histories of the Second Wave of Feminism: summarised from interviews by Viv Honeybourne and Ilona Singer*, vols. 1 and 2 (Bristol: Feminist Archive South), p. 26. http://feministarchivesouth.org.uk/wp-content/uploads/2013/02/Personal-Histories-of-the-Second-Wave-of-Feminism.pdf, accessed 6 July 2017; Bruley, ‘Women’s Liberation’, p. 729.

49 *Bristol Women’s Liberation Newsletter*, September 1973, Ellen Malos Archive, Feminist Archive South, University of Bristol Library Special Collections, Bristol (DM2123/8/115), hereafter: *Newsletter*.

50 Ibid. January 1974; April 1974.

51 Betty Underwood, ‘What Makes Pregnancy Tests So Difficult?’, *Morning Star*, 16 June 1976.

52 ‘The Free Pregnancy Testing Service’, November 1974 (DM2123/8/115).

53 Michael Litchfield & Susan Kentish (1974) *Babies for Burning: the abortion business in Britain* (London: Serpentine); Colin Francome (1980) *Social Forces and the Abortion Law* (Ph.D. thesis, Middlesex Polytechnic), pp. 241–245.

54 *Newsletter*, April 1975.

55 Ibid.

56 Murphy, *Seizing the Means*. See also Katie Ann Hasson (2012) Making Appropriation ‘Stick’: stabilizing politics in an ‘inherently feminist’ tool, *Social Studies of Science*, 42, pp. 638–661. For a more general discussion: Ron Eglash, Jennifer L. Croissant, Giovanna di Chiro & Rayvon Fouché (Eds) (2004) *Appropriating Technology: vernacular science and social power* (Minneapolis: University of Minnesota Press).

57 Author’s interview.

58 For a recent discussion of skills in medical practice: Nicholas Whitfield & Thomas Schlich (2015) Skills Through History, *Medical History*, 59, pp. 349–360.

59 Author’s interview; Ellen Malos (2004) He Always Says He’s Sorry Afterwards, in Helene Curtis & Mimi Sanderson (Eds), *The Unsung Sixties: memoirs of social innovation* (London: Whiting & Birch), pp. 284–297, p. 286. One American service was set up in the late 1960s by two female laboratory technicians: Leslie J. Reagan (2000) Crossing the Border for Abortions: California activists, Mexican clinics, and the creation of a feminist health agency in the 1960s, *Feminist Studies*, 26, pp. 323–348, 338.

60 For example, Keir Waddington (2010) More Like Cooking than Science: narrating the inside of a British medical laboratory, 1880–1914, *Literature and Science*, 3, pp. 50–70. On the subsequent domestication of home test kits: Janet E. Childerhouse & Margaret E. MacDonald (2013) Health Consumption as Work: the home pregnancy test as a domesticated health tool, *Social Science & Medicine*, 86, pp. 1–8.

61 See Leathard, *The Fight for Family Planning*, p. 176.

62 C.P.A.G., ‘How We Set Up’.

63 Kingsgate Place Diary no 4, 25 April 1974, Papers of Tamar Karet, Women’s Library @ LSE, London (7TAK/03).

64 73% of British households had a refrigerator in 1972: Ann Bridgwood (2000) *Living in Britain: results from the 1998 general household survey* (London: H.M.S.O.), p. 6.

65 Kingsgate Place Diary no 4, 25 April 1974 (7TAK/03).

66 *Newsletter*, November 1975; Refrigerator space was also a scarce resource in America: Anne Finn Enke (2007) *Finding the Movement: sexuality, contested space, and feminist activism* (Durham NC: Duke University Press), p. 78.

67 Meeting of Pregnancy Testers, Bristol, 11 July 1975 (DM2123/8/115).

68 Robert Howard & Frances Dunkley (1977) An Introduction to the Many Faces of Pregnancy Testing (Sheffield: BPAS), FPA records, Wellcome Library, London (SA/FPA/C/B/2/30/1), § 7.1.3.

69 Malos, ‘He Always Says He’s Sorry’, p. 287.

70 *Newsletter*, October 1978. See also September 1980.

71 Ruth Wallsgrove, ‘The Nuclear Threat’, *Spare Rib*, 112, November 1981, pp. 3–4.

72 ‘Talking with Women in Bristol’, p. 35.

73 Underwood, ‘What Makes Pregnancy Tests So Difficult?’.

74 Interview with Sue Jones.

75 Towards a Politics of Pregnancy Testing, October 1976 (GBR/0271/GCIP CWLA).

76 C.P.A.G.—Pregnancy Testing and Contraceptive Advice, leaflet (GBR/0271/GCIP CWLA).

77 Women’s Centre Pregnancy Testing, W.A.C.C., Bristol, *c*.1973 (DM2123/8/115).

78 Dr G. M. Clayton to David Ennals, 7 February 1979, Pregnancy: immunological tests; reassessing regulation, Ministry of Health records, National Archives, Kew (MH156/278).

79 Records of the Brighton Women’s Centre, Women’s Library @ LSE (5BWC/survey/G).

80 C.P.A.G., ‘How We Set Up’.

81 Howard & Dunkley, ‘An Introduction’, § 5.4. See also Lara V. Marks (2001) *Sexual Chemistry: a history of the contraceptive pill* (New Haven CT: Yale University Press); Hera Cook (2004) *The Long Sexual Revolution: English women, sex, and contraception 1800–1975* (Oxford: Oxford University Press).

82 Meeting of Pregnancy Testers, Bristol, 11 July 1975.

83 ‘Pregnancy testers’ meeting’, Thursday, 17 February 1976, at the Women’s Centre, Bristol.

84 Interview with Sue Jones.

85 Paintin, *Abortion Law Reform*, p. 71.

86 Michèle Roberts (2007) *Paper Houses: a memoir of the ’70s and beyond* (London: Virago), pp. 136–137.

87 Records of the Brighton Women’s Centre (5BWC/survey/G). Women seeking abortion were typically referred to Wistons, the ‘first BPAS nursing home’, which had opened in 1971: Suzanne Mackenzie (1989) *Visible Histories: women and environments in a post-war British city* (Montreal: McGill-Queen’s University Press), pp. 70–72.

88 C.P.A.G., ‘How We Set Up’.

89 Helen Seed, Bristol Women’s Centre Free Pregnancy Testing Service, 13 September 1974 (DM2123/8/115).

90 Howard & Dunkley, ‘An Introduction’, § 6.5.

91 Honeybourne & Singer, *Personal Histories of the Second Wave*, p. 26.

92 Interview with Sue Jones. See also Naomi Pfeffer (1993) *The Stork and the Syringe: political history of reproductive medicine* (Cambridge: Polity Press).

93 Nancy MacKeith (1978) *The New Women’s Health Handbook* (London: Virago), p. 25.

94 Stevienna Marie de Saille (2012) *From ‘Death of the Female’ to ‘Life Itself’: a socio-historic examination of FINRRAGE* (Ph.D. thesis, University of Leeds), p. 2. See also Pfeffer (1985) Not So New Technologies, *Trouble and Strife*, 5, pp. 46–50.

95 ‘Oxford, the Concise Women’s Guide’, *Spare Rib*, 55, February 1977, p. 46. On LIFE pregnancy testing and counsellors: David Marsh & Joanna Chambers (1981) *Abortion Politics* (London: Junction), pp. 60–61.

96 *Spare Rib*, 60, July 1977, p. 52.

97 *Spare Rib*, 67, February 1978, p. 24; Bruley, ‘Women’s Liberation’, p. 730.

98 Kate Holman, ‘Self Help Blocked’, *Spare Rib*, 83, June 1979, p. 12. The Balham Family Centre also started pregnancy testing in 1978: Ernest Rodker, ‘Sonia Markham Obituary’, *the Guardian*, 17 March 2016, www.theguardian.com/science/2016/mar/17/sonia-markham-obituary, accessed 12 September 2016.

99 Jill Nicholls, ‘Going Back to the Beginning’, *Spare Rib*, 47, June 1976, p. 27.

100 *Newsletter*, June 1977.

101 Ibid.; *Spare Rib*, 70, May 1978, p. 24; 107, June 1981, pp. 28–29.

102 Carolyn Faulder, Christine Jackson & Mary Lewis, *The Women’s Directory* (London: Virago), p. 198.

103 MacKeith, *New Women’s Health Handbook*, p. 63.

104 Angela Phillips & Jill Rakusen (1978) *Our Bodies, Ourselves* (London: Penguin), p. 350.

105 Carolyn Faulder (1978) *Talking to your Doctor* (London: Virago), p. 34.

106 ‘The Free Pregnancy Testing Service’, November 1974 (DM2123/8/115).

107 *Newsletter*, November 1976.

108 ‘Pregnancy Testing’, December 1979 (DM2123/8/115).

109 Many other locally organised activities similarly persisted: Bruley, ‘Women’s Liberation’, p. 735. On the conference: Jeska Rees (2010) A Look Back at Anger: the women’s liberation movement in 1978, *Women’s History Review*, 19, pp. 337–356.

110 Honeybourne & Singer, *Personal Histories of the Second Wave*, p. 5.

111 *Newsletter*, October–November 1983.

112 ‘Pregnancy testing’, *Spare Rib*, 122, September 1982 p. 32.

113 ‘Brent Women’s Pregnancy Testing Group’, *Spare Rib*, 236, July 1992, p. 57.

114 Records of the Brighton Women’s Centre (5BWC/survey/G).

115 Author’s interview.

116 Peggy Foster (1989) Improving the Doctor/Patient Relationship: a feminist perspective, *Journal of Social Policy*, 18, pp. 337–361; Rosemary Pringle (1998) *Sex and Medicine: gender, power and authority in the medical profession* (Cambridge: Cambridge University Press), pp. 204–205; Joy Merrell (2000) ‘You Don’t Do it for Nothing’: women’s experiences of volunteering in two community well woman clinics, *Health and Social Care in the Community*, 8, pp. 31–39.

117 Author’s interview.

118 Jesse Olszynko-Gryn (Forthcoming) Thin Blue Lines: product placement and the drama of pregnancy testing in British cinema and television, *British Journal for the History of Science*, 50.

119 The speculum, a visually striking object, was prominently wielded by Wonder Woman on the cover of *Sister*, the Los Angeles feminist newspaper, in 1973: Donna J. Haraway (1997) The Virtual Speculum in the New World Order, *Feminist Review*, 55, pp. 22–72. Today, they can be ordered online from the Beautiful Cervix Project, a boutique supplier: http://beautifulcervix.com, accessed 12 September 2016.

120 See Leathard, *Fight for Family Planning*, p. 73; Cohen, ‘Private Lives’, p. 98; Pfeffer, *Stork and the Syringe*, pp. 59–60; Martin Richards (2008) Artificial Insemination and Eugenics: celibate motherhood, eutelegenesis and germinal choice, *Studies in History and Philosophy of Biological and Biomedical Science*s, 39, pp. 211–221.

121 Bruley, ‘Women’s Liberation’, p. 733.

122 Rose Reeve, of the Archway group, particularly remembered ‘an Asian couple’ who likely found out about the service from an advertisement in the local papers: Reeve, email to author, 3 May 2016.

123 Alondra Nelson (2011) *Body and Soul: The Black Panther Party and the fight against medical discrimination* (Minneapolis: University of Minnesota Press), p. 116. At least a few Black British feminists were involved in pregnancy testing: Interview with Donna Jackman and Carol Leeming, March 2009, Oral Histories of the Black Women’s Movement: The Heart of Race, Black Cultural Archives, London (ORAL/1/8).

124 Steven Epstein (1996) *Impure Science: AIDS, activism, and the politics of knowledge* (Berkeley: University of California Press), p. 231.

125 See Cohen, ‘Private Lives’, pp. 108–111; Simon Szreter & Kate Fisher (2010) *Sex Before the Sexual Revolution: intimate life in England, 1918–1963* (Cambridge: University of Cambridge Press), p. 254.

126 Bruley, ‘Women’s Liberation’, p. 729.

127 On the limits of privacy in home pregnancy testing: Linda L. Layne (2010) Why the Home Pregnancy Test Isn’t the Feminist Technology It’s Cracked Up to Be and How to Make it Better, in Linda L. Layne, Sharra L. Vostral & Kate Boyer (Eds) *Feminist Technology* (Urbana: University of Illinois Press), pp. 89–118.

128 See Cohen, ‘Private Lives’, p. 99.

129 For a discussion of the feminist potential of home pregnancy testing that resonates with many of the themes raised in this article: Layne, ‘Why the Home Pregnancy Test isn’t the Feminist Technology It’s Cracked Up to Be’.

